# Supercritical Phase Inversion: A Powerful Tool for Generating Cellulose Acetate-AgNO_3_ Antimicrobial Membranes

**DOI:** 10.3390/ma13071560

**Published:** 2020-03-28

**Authors:** Lucia Baldino, Stefano Cardea, Ernesto Reverchon

**Affiliations:** Department of Industrial Engineering, University of Salerno, Via Giovanni Paolo II, 132, 84084 Fisciano (SA), Italy; lbaldino@unisa.it (L.B.); scardea@unisa.it (S.C.)

**Keywords:** cellulose acetate, silver nitrate, membranes, supercritical phase inversion, supercritical CO_2_

## Abstract

Antimicrobial composite membranes, formed by cellulose acetate loaded with AgNO_3_ particles, were produced by supercritical phase inversion. Different cellulose acetate concentrations were tested (15%, 20%, 30%(*w*/*w*)), whereas the active agent (i.e., silver nitrate) concentration was fixed at 0.1%(*w*/*w*) with respect to the quantity of polymer used. To determine the influence of the process parameters on membranes morphology, the pressure and temperature were varied from 150 to 250 bar and from 55 to 35 °C, respectively. In all cases, regularly porous membranes were produced with a uniform AgNO_3_ distribution in the membrane matrix. Silver release rate depended on membrane pore size, covering a time interval from 8 to 75 h.

## 1. Introduction

Active polymer nanocomposites are a relevant research field in nanotechnology [1,2,3], since they combine the polymer characteristics (e.g., mechanical properties, biocompatibility, low cost, etc.) with the active role of the loaded compounds (e.g., antimicrobial, antioxidant, etc.). They can be used in different applications, depending on their properties, e.g., antimicrobial devices are widely used in medicine [4], pharmaceutical [5] and food [6] fields, in form of film [7], membranes [8] and particles [9].

Silver powder is frequently used as an antimicrobial agent [10,11,12,13]. In particular, ionic silver interacts with thiol groups of enzymes, inactivating them [14]. Therefore, the interest in nanocomposites loaded with silver nanoparticles is due to the antimicrobial effect of nanosilver that is maximized when a homogeneous dispersion of this active compound on the surface and inside the polymer host is assured [15].

Numerous authors have produced polymer devices loaded with silver, using different techniques. Miranda et al. [16] prepared silver-hydroxyapatite nanocomposites at 1%(*w*/*w*) of metallic silver, obtaining a logarithmic reduction larger than 20 of *Escherichia coli* and *Micrococcus luteus*. Shuai et al. [17] proposed an in-situ growth of silver in mesoporous bioactive glass to obtain a scaffold with antibacterial activity; these loaded scaffolds were produced by laser sintering. The antibacterial tests showed a bacterial inhibition rate of more than 99% against *Escherichia coli*, and, at the same time, good cytocompatibility in facilitating osteoblast adhesion and proliferation. Ito et al. [18] produced an antimicrobial coating material by embedding silver nanoparticles in polymer nanosheets, composed of layers of chitosan and sodium alginate, using a photo-reduction method. Girdthep et al. [19] developed biodegradable polymer nanocomposites by compression molding, to be used in the packaging of moisture-sensitive products. Poly (lactic acid) was the main component of the nanocomposites with poly (butylene adipate-co-terephthalate) as the flexibility enhancer. Silver-loaded kaolinite was also incorporated into the compatibilized blends. These nanocomposites exhibited a water vapor permeability reduction of about 42%. Shao et al. [20] produced, by freeze-drying, cellulose/sodium alginate composites, loaded with silver sulfadiazine, for wound dressings. These composites showed excellent antibacterial activity and good biocompatibility.

Even if these nanocomposites were characterized by antimicrobial properties useful for different applications, they were produced by complex processes that are expensive, time consuming, and with a reduced control over the polymeric device morphology. Moreover, the uniform silver distribution inside the polymeric matrix was only checked in some cases, and this is one of the relevant requisites to favor a correct performance of the antimicrobial device.

Supercritical CO_2_ (SC-CO_2_)-assisted phase inversion has been proposed as an alternative to overcome these limitations, producing regular porous membranes of various polymers that, in some cases, were also loaded with active agents [21,22,23,24,25,26]. Using this technique, the organic solvent, required for sample preparation, is completely and rapidly removed from the final membrane thanks to the chemical affinity with SC-CO_2_. Moreover, the supercritical mixture (CO_2_ and solvent) is characterized by negligible surface tension and gas-like diffusivity, and avoids porous structure collapse and active compounds deposition/separation in some regions of the polymeric matrix. This process also allows for control of the membranes morphology that can be modulated in terms of porosity and pore size, changing the operative conditions (i.e., mainly pressure and temperature). Baldino et al. [24] produced biodegradable cellulose acetate and curcumin nanostructured membranes using this supercritical process, testing different parameters: 12–24%(*w*/*w*) cellulose acetate, 10–20%(*w*/*w*) curcumin, 35–55 °C, 150–250 bar. These authors obtained a uniform distribution at nanometric level of curcumin and produced solvent-free porous membranes (5 ppm acetone residue). In another work, Baldino et al. [26] incorporated silver-loaded hydroxyapatite nanoparticles into poly (vinyl alcohol) membranes. Solutions at 20%(*w*/*w*) PVA produced membranes with cellular morphology and nanoporous pore walls, whereas, 30% and 50%(*w*/*w*) PVA solutions produced nanostructured membranes. They showed a significant *E. coli* inhibition, at an Ag concentration of 9 ppm.

In this work, biocompatible and biodegradable membranes of cellulose acetate (CA), loaded with AgNO_3_ particles, were produced by SC-CO_2_ assisted phase inversion for antimicrobial applications. A systematic physico-chemical characterization of these loaded membranes was performed, to verify the process influence on membranes morphology and pore size, and silver dispersion into these active devices, with the aim of optimizing the active agent release and the effectiveness of these antimicrobial composite membranes.

## 2. Materials and Methods

Cellulose acetate, CA, (average Mn ca. 50000 with acetyl content of 39.7%), acetone (purity 99.5%) and AgNO_3_ powder, were bought from Sigma-Aldrich (Milan, Italy). CO_2_ (purity 99%) was purchased from Morlando Group S.R.L. (Sant’Antimo (NA), Italy). All materials were processed as received.

Solutions were prepared by dissolving CA at 15%, 20% and 30%(*w*/*w*) in acetone at 35 °C and 100 rpm for 12 h. Then, AgNO_3_ powder, at 0.1%(*w*/*w*) with respect to CA, was added to the polymer solutions, left to mixing for other 2 h at 35 °C and 100 rpm. This AgNO_3_ loading was selected according to the literature, to guarantee a proper Ag amount against *Escherichia coli* and *Staphylococcus aureus* [27,28,29]. The obtained suspensions were distributed on stainless steel caps, having a diameter of 2 cm and a thickness of about 1 mm.

These caps were rapidly put inside the high-pressure vessel (a 316 stainless steel vessel with an internal volume of 200 mL) to minimize evaporation of the solvent. The vessel was closed and filled with SC-CO_2_ up to the operative pressure, using a high-pressure pump (mod. LDB1, Lewa, Passirana di Rho (MI), Italy). Pressure in the vessel was measured by a test gauge (mod. MP1, OMET, Pessano Con Bornago (MI), Italy) and regulated using a micrometering valve (mod. 1335G4Y, Hoke, Spartanburg, SC, USA). Temperature was regulated using PID controllers (mod. 305, Watlow, Milan, Italy). At the exit of the vessel, a rotameter (mod. D6, ASA, Sesto San Giovanni (MI), Italy) was used to measure CO_2_ flow rate that was regulated at 1.5 kg/h.

The membranes were cryo-fractured using liquid nitrogen (SOL, Milan, Italy), then, samples were sputter-coated with gold (Agar Auto Sputter Coater mod. 108 A, Stansted, UK) at 30 mA for 120 s and analyzed by FESEM (field emission scanning electron microscope, mod. LEO 1525, Carl Zeiss SMT AG, Oberkochen, Germany) to study membrane morphology. Sigma Scan Pro 5.0 (Jandel Scientific, San Rafael, Canada) and Origin 8.5 (Microcal, Northampton, USA) were used to determine the average diameter of membrane pores. Images taken at various locations in the membrane section were used for each calculation. About 300 pores for each sample were measured.

Porosity measurements were performed on the composite membranes using an Ultrapycnometer 1000 (Quantachrome instruments, Anton Paar Italy S.R.L., Rivoli (TO), Italy). Five samples for each process condition were analyzed; therefore, the obtained porosity values were the mean of the porosities measured on these samples, with the corresponding standard deviation, as indicated in [30].

Acetone residue in the final composite membranes was measured by a headspace (HS) sampler (mod. 7694E, Hewlett Packard, USA) coupled to a gas chromatograph (GC) interfaced with a flame ionization detector (GC-FID, mod. 6890 GC-SYSTEM, Hewlett Packard, Palo Alto, CA, USA), using the method described in [24].

DSC (DSC 30 Mettler, Toledo, Spain) was carried out to identify any changes in the thermograms of polymer/active compound membranes compared to the ones of pure substances. A calorimetric analysis was performed in the temperature range between 0 and 300 °C, with a heating rate of 10 °C/min, using nitrogen as inert gas, at a flow rate of 50 L/min.

Membranes were analyzed by energy dispersive X-ray spectroscopy (EDX INCA Energy 350, Oxford Instruments, High Wycombe, UK,) previously cryo-fractured using liquid nitrogen and sputter-coated with chromium (EMITECH K575X peltier-cooled), in order to control the dispersion of AgNO_3_ in the polymer matrix.

Permeability tests were performed using a Quantachrome 3Gz porometer (Anton Paar Italy S.R.L., Rivoli (TO), Italy) and selecting nitrogen as the inert gas.

Ag release kinetics were determined measuring the increase of the active compound concentration in 25 mL of distilled water and at room temperature, using a Varian (mod. Cary® 50, Palo Alto, CA, USA) UV/Vis spectrophotometer. After the composite membrane immersion in water, the system was stirred at 50 rpm, to favor the aqueous medium penetration in the membrane and to assure a homogeneous dispersion of the released Ag in water, in order to read a correct absorbance value. The measurements were performed at 420 nm; i.e., the wavelength at which Ag shows maximum absorption [31].

## 3. Results and Discussion

CA membrane production by SC-CO_2_ assisted phase inversion was successfully performed and studied in a previous paper by the same research group [32]. The realization of new AgNO_3_ loaded CA membranes was tried in this work.

The first set of experiments was focused on the effect of process kinetics and thermodynamics on the membrane’s morphology. Pressure/temperature combination was changed in the range 150 bar 55 °C (ρ_CO2_ ≈ 0.65 g/cm^3^) and 250 bar 35 °C (ρ_CO2_ ≈ 0.90 g/cm^3^) to determine the operative conditions suitable to obtain a regular porous membranes morphology, as it is required for a controlled silver release. Indeed, it is known from the literature that SC-CO_2_ density influences the kinetics and thermodynamics of the supercritical phase inversion, and, consequently, membrane morphology and pore size [33].

**Figure 1:**
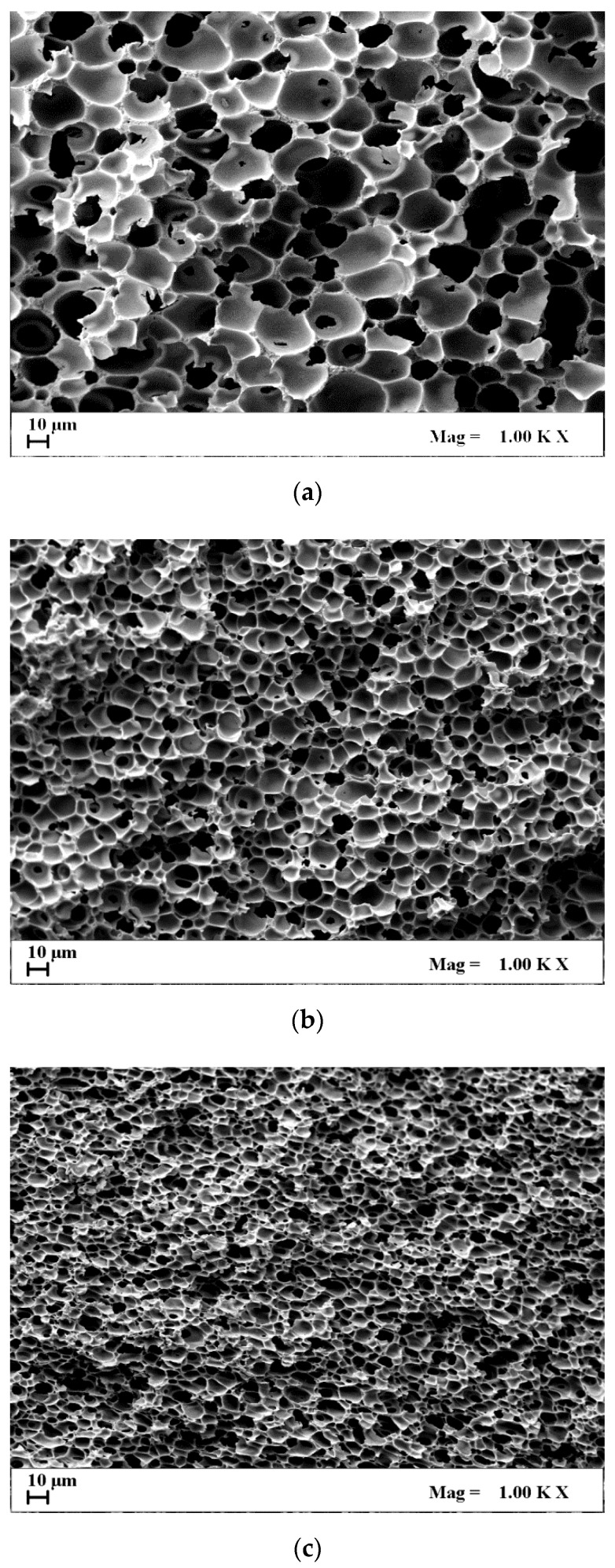
SEM images of AgNO_3_-loaded CA membranes, at 30%(*w*/*w*) CA, produced at: (**a**) 150 bar, 55 °C; (**b**) 200 bar, 45 °C; (**c**) 250 bar, 35 °C.

In SEM images of Figure 1a–c, internal sections of 30%(*w*/*w*) CA membranes loaded with AgNO_3_, obtained at different operative conditions, were reported. Increasing SC-CO_2_ solvent power (from Figure 1a–c), due to SC-CO_2_ density increase, the membrane’s morphology did not change, but porosity and pore size decreased. In particular, passing from 150 bar 55 to 250 bar 35 °C, porosity decreased from about 84% to 81% and mean pores size decreased from about 10 to 7 μm (see Table 1). To explain this result, we can refer to the theory of the phase inversion process: increasing the SC-CO_2_ density means that an increase in the phase inversion rate is obtained, too. When the phase inversion is faster, the polymer lean-phase (i.e., SC-CO_2_ and solvent), after the nucleation step, has less time to grow; i.e., smaller pores are obtained after solvent extraction [34].

Once ascertained that operative conditions variation for this polymeric system only produced a mean pore size reduction, the polymer concentration was decreased from 30% to 15%(*w*/*w*); AgNO_3_ powder amount was always fixed at 0.1%(*w*/*w*) with respect to CA content. The aim was to find the best balance between a porous membrane morphology (changing the operative conditions) and the minimum amount of polymer required to obtain this result.

**Figure 2:**
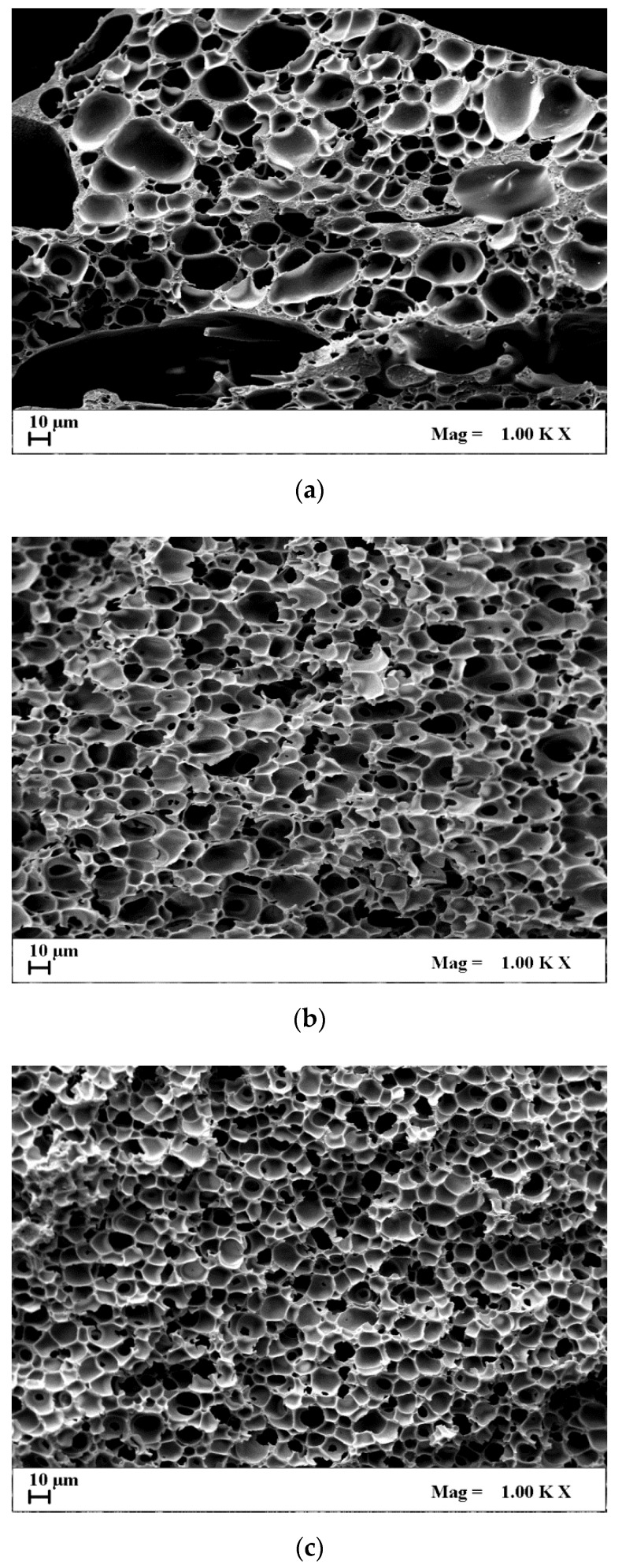
SEM images of AgNO_3_-loaded CA membranes, produced at 200 bar and 45 °C: (**a**) 15%(*w*/*w*) CA, (**b**) 20%(*w*/*w*) CA, (**c**) 30%(*w*/*w*) CA.

SEM images of Figure 2a–c show the effect of polymer concentration on CA/AgNO_3_ composite membranes internal section morphology, obtained at 200 bar and 45 °C. The first observation is that cellular morphologies were obtained in all cases, and no AgNO_3_ particles accumulation along the membrane structure was found. This result was obtained at all process conditions tested in this work. However, the addition of a solute (such as AgNO_3_) in the starting polymeric solution could theoretically change the thermodynamics and the kinetics of the process, behaving as a nucleating agent for pore formation, or reducing the phase inversion rate [35]. The main drawback can be the solute accumulation on the bottom of the membrane, as in the traditional phase inversion [36,37]. In this case, the addition of AgNO_3_ did not affect the supercritical process, since a regular cellular structure was obtained along the membrane section, and the presence of particle aggregates was not detected.

Moreover, CA increase from 15% to 30%(*w*/*w*) favored a decrease in membrane porosity and pore size. In particular, at 15%(*w*/*w*) CA, some macrovoids were present, as shown in Figure 2a, and the pores surrounding the macrovoids had a mean diameter of about 12.7 ± 3.7 μm. The overall porosity was about 87%, whereas, at 20% and 30%(*w*/*w*) CA, a homogeneous cellular morphology was generated, with a pore size of 9.8 ± 2.9 μm and 8.4 ± 1.8 μm, and a porosity of 84.2% and 82.4%, respectively. All data measured for the composite membranes produced in this work are reported and compared in Table 1.

Increasing the polymer concentration in the starting solution, the demixing point inside the miscibility gap of the ternary system CA/acetone/SC-CO_2_ changes in the direction of the polymer vertex, favoring the formation of smaller pores during the nucleation and growth of the polymer lean-phase [31]. In general, the larger the polymer concentration is in the starting solution (cellular morphology is favored), the smaller the pores are expected to be (producing a more compact porous membrane, i.e., less porous). The formation of macrovoids is obtained, instead, at lower polymer concentrations (i.e., 15%(*w*/*w*) CA in this specific system), due to the same reason previously explained, related to a lower solution viscosity [31].

Analyses of the solvent residue were also performed on the composite CA membranes, as described in Materials and Methods. Acetone residue was found to be lower than 5 ppm, in all cases and at all process conditions tested. Therefore, the supercritical assisted phase inversion confirmed to be a successful process in extracting all the organic solvent from the polymeric matrix when the operative conditions are properly selected, making these composite membranes safe for various applications.

**Figure 3:**
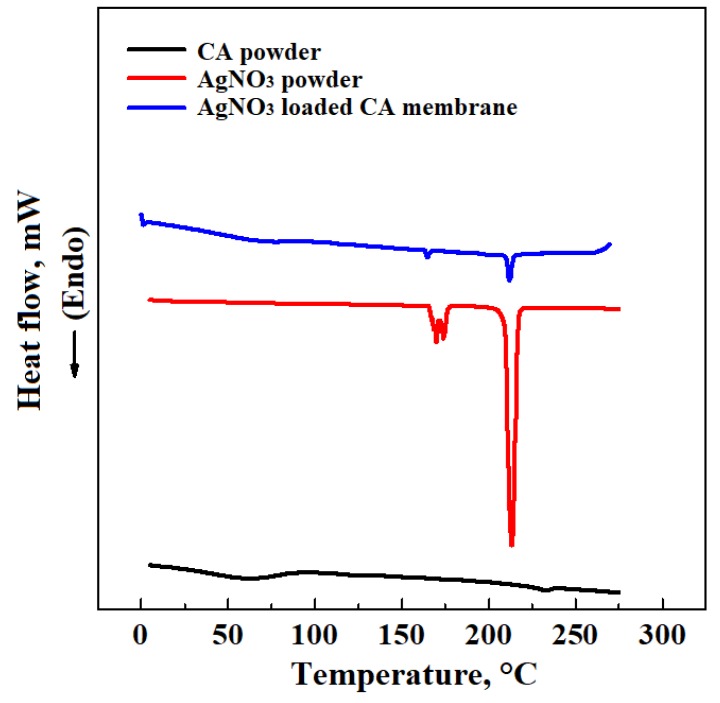
Comparison among AgNO_3_, CA and AgNO_3_/CA composite membranes thermograms.

In order to verify the correct loading and dispersion of AgNO_3_ particles into CA membranes, DSC and EDX analyses were performed. Figure 3 shows the thermograms obtained from the DSC analysis of AgNO_3_, CA and AgNO_3_/CA composite membranes (at 20%(*w*/*w*) CA and produced at 200 bar and 45 °C), performed according to the procedure described in Materials and Methods. The DSC results show evidence that the silver salt had a melting peak at about 215 °C, whereas CA had the glass transition temperature at 173 °C, and the melting peak at about 230 °C. These temperature values of the two components are consistent with the literature [38,39], and also characterize the thermogram of the composite membrane. Therefore, DSC analysis confirmed the AgNO_3_ encapsulation in the polymeric matrix.

**Figure 4:**
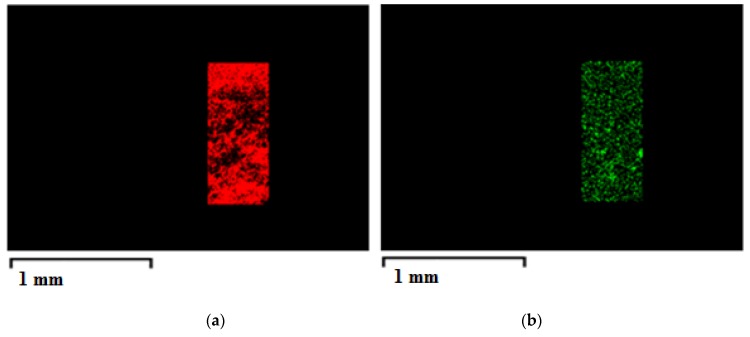
EDX maps of a CA/AgNO3 composite membrane internal section: (**a**) C atoms; (**b**) Ag atoms.

Relevant information to be verified is the AgNO_3_ dispersion into the CA membrane for two main reasons. The first one is related to the effectiveness of the supercritical process in avoiding the active agent accumulation in some regions of the membrane; the second one influences the antimicrobial membrane performance, since it has to guarantee the same effect whichever is the membrane surface in contact with the undesired host. Figure 4 shows the EDX maps of a composite membrane internal section, at 20%(*w*/*w*) CA and 0.1%(*w*/*w*) AgNO_3_. In particular, carbon atoms were selected for polymer identification and Ag atoms for AgNO_3_ powder. As clearly represented in these EDX maps, Ag distribution practically overlapped the carbon matrix, i.e., a uniform AgNO_3_ powder distribution was obtained in the composite membrane. This result is mainly due to supercritical process [26]. Indeed, since the SC-CO_2_ assisted phase inversion is faster with respect to the traditional one, the aggregation and the solute cluster formation during the process is avoided, freezing the starting active compound distribution inside the polymeric system.

In the last part of this work, the performance of these CA composite membranes, depending on their morphology and pore size, was studied. In particular, nitrogen permeability tests, and Ag release tests were performed.

**Figure 5:**
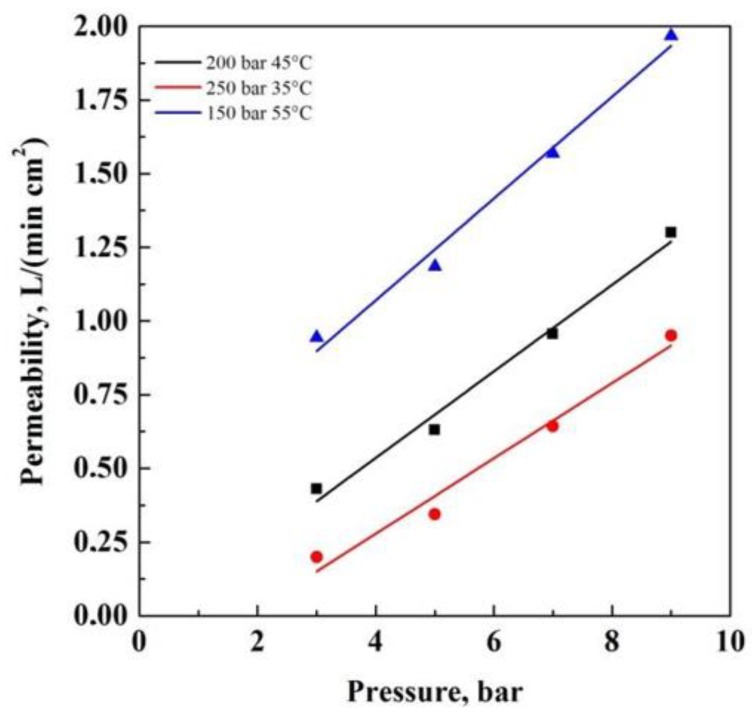
Nitrogen permeability tests performed on 20%(*w*/*w*) CA membranes with 0.1%(*w*/*w*) AgNO_3_, obtained at 150 bar 55 °C, 200 bar 45 °C, 250 bar 35 °C.

Figure 5 reports the permeability tests performed on 20%(*w*/*w*) CA composite membranes, obtained at different SC-CO_2_ solvent powers. These membranes were selected as an intermediate case among the produced membranes in this work. The aim was to put in evidence the effect of the membrane pore size on the gas flow rate. Increasing the SC-CO_2_ solvent power (from 150 bar 55 °C, to 250 bar and 35 °C) to carry out the supercritical process, the nitrogen permeability corresponding to the obtained membranes decreases. This result can be related to the morphology of the membranes: when a larger SC-CO_2_ solvent power was used during processing, the obtained membranes presented smaller pores (see Figure 1 and Table 1); consequently, the gas flow rate across these membranes was lower, since it faced an increased pressure drop.

**Figure 6:**
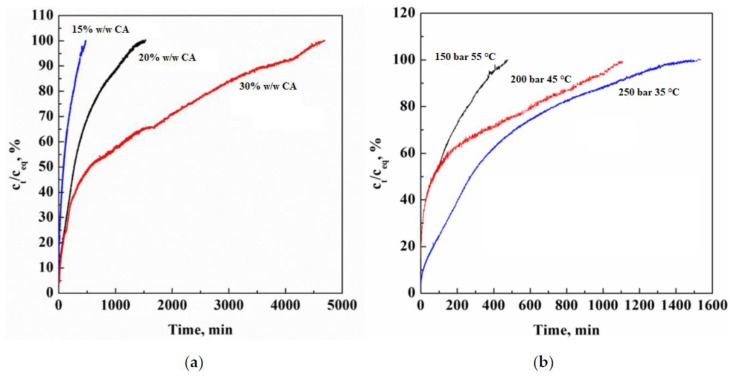
Ag release curves from CA membranes: (**a**) at different CA concentrations; (**b**) produced at different process operative conditions.

In Figure 6a,b, Ag release test curves from the CA membranes, obtained at different CA starting concentration and operative conditions, are reported. Ag concentration measured (c_t_) was normalized on the Ag concentration at equilibrium (c_eq_; i.e., the maximum amount of Ag released and detected in each analysis) versus time. The analyses of the Ag release curves reported in Figure 6a, related to the CA composite membranes obtained at different polymer concentrations (from 15% to 30%(*w*/*w*)), and produced at 250 bar and 35 °C, shows the effect of the membrane pore size on the Ag release rate. Increasing the polymer concentration, a pore size decrease was obtained and, therefore, a slower Ag release rate was induced, reaching the maximum released Ag concentration after 75 h. On the contrary, the composite membranes obtained at lower polymer concentration (i.e., 15%(*w*/*w*)) showed the maximum released Ag concentration after 8 h. Moreover, a burst effect can be detected in the first minutes of the analysis. This behavior can be explained considering that, at 15%(*w*/*w*) CA, macrovoids formation in the membrane was observed (Figure 2a). These macrovoids favored a fast Ag release due to an increased mass transfer of the simulant medium (i.e., water) inside and outside the membrane, according to a diffusion mechanism [40]. The membrane at 20%(*w*/*w*) CA presented an intermediate behavior, releasing Ag in about 25 h. This result can be explained considering that 20%(*w*/*w*) CA membrane was characterized by a homogeneous porous structure, and, as a consequence, produced a higher mass transfer resistance to the simulant medium, with respect to the 15%(*w*/*w*) CA membranes, but lower with respect to the 30%(*w*/*w*) CA membranes.

Ag release behavior depends also on the process operative conditions used for CA membranes production, as reported in Figure 6b. Again, membranes morphology and pores size determined different Ag release rates. The composite membranes obtained at higher SC-CO_2_ solvent power (i.e., 250 bar and 35 °C) induced a slower Ag release, completing the release of the active compound in about 25 h, due to the smaller pores (Figure 1c). On the contrary, the composite membranes obtained at 150 bar and 55 °C presented larger pores that favored the Ag release: the maximum Ag release was observed in about 8 h.

## 4. Conclusions and Perspectives

Supercritical phase inversion confirmed to be a flexible process to produce porous loaded membranes. The membrane mean pore size changed varying SC-CO_2_ density and, consequently, the thermodynamics and kinetics of the process. Operating in this manner, a uniform active agent distribution inside the CA membrane matrix was assured and the silver release rate was modulated depending on the mass transfer resistance opposed by the membrane structure. A continued release of silver in water was measured up to 75 h (i.e., 3 days) for the CA membranes characterized by smaller pores. On the other hand, when a rapid action of the active principle is required (i.e., to face media that exhibit a large contaminants charge), a faster release rate can be obtained, in about 8 h, selecting the proper composite membrane. Therefore, these membranes can be used for different antimicrobial applications, from water treatment to food shelf-life increase, since they are solventless, biocompatible and biodegradable. In future, in vitro antimicrobial tests against Gram-microorganism can be tried, in order to confirm their antimicrobial activity dependent on the Ag release rate for a real application.

## Figures and Tables

**Table 1:** Mean pore size and porosity of all the CA membranes loaded with AgNO_3_, produced in this work.

CA Concentration (AgNO_3_ 0.1%(*w*/*w*) CA)	Mean Pore Diameter, μm	Porosity, %
**150 bar 55 °C**		
15%(*w*/*w*)	14.36 ± 4.05	89.7 ± 6.2
20%(*w*/*w*)	10.93 ± 2.54	85.6 ± 5.4
30%(*w*/*w*)	10.06 ± 2.19	84.1 ± 4.7
**200 bar 45 °C**		
15%(*w*/*w*)	12.67 ± 3.66	87.4 ± 5.8
20%(*w*/*w*)	9.84 ± 2.93	84.2 ± 4.9
30%(*w*/*w*)	8.42 ± 1.78	82.4 ± 4.2
**250 bar 35 °C**		
15%(*w*/*w*)	8.73 ± 2.14	83.7 ± 4.6
20%(*w*/*w*)	7.99 ± 1.79	82.3 ± 4.1
30%(*w*/*w*)	6.86 ± 1.45	81.2 ± 3.8
